# How selective antagonists and genetic modification have helped characterise the expression and functions of vascular P2Y receptors

**DOI:** 10.1007/s11302-024-10016-z

**Published:** 2024-05-13

**Authors:** Markie O. Dales, Robert M. Drummond, Charles Kennedy

**Affiliations:** Strathclyde Institute of Pharmacy & Biomedical Sciences, University of Strathclyde, 161 Cathedral Street, Glasgow, G4 0RE UK

**Keywords:** AR-C118925XX, Atherosclerosis, Cangrelor, Hypertension, MRS2179, MRS2578, NF340, P2Y receptor, PPTN, Vasoconstriction, Vasodilation

## Abstract

Vascular P2Y receptors mediate many effects, but the role of individual subtypes is often unclear. Here we discuss how subtype-selective antagonists and receptor knockout/knockdown have helped identify these roles in numerous species and vessels. P2Y_1_ receptor-mediated vasoconstriction and endothelium-dependent vasodilation have been characterised using the selective antagonists, MRS2179 and MRS2216, whilst AR-C118925XX, a P2Y_2_ receptor antagonist, reduced endothelium-dependent relaxation, and signalling evoked by UTP or fluid shear stress. P2Y_2_ receptor knockdown reduced endothelial signalling and endothelial P2Y_2_ receptor knockout produced hypertensive mice and abolished vasodilation elicited by an increase in flow. UTP-evoked vasoconstriction was also blocked by AR-C118925XX, but the effects of P2Y_2_ receptor knockout were complex. No P2Y_4_ receptor antagonists are available and P2Y_4_ knockout did not affect the vascular actions of UTP and UDP. The P2Y_6_ receptor antagonist, MRS2578, identified endothelial P2Y_6_ receptors mediating vasodilation, but receptor knockout had complex effects. MRS2578 also inhibited, and P2Y_6_ knockout abolished, contractions evoked by UDP. P2Y_6_ receptors contribute to the myogenic tone induced by a stepped increase in vascular perfusion pressure and possibly to the development of atherosclerosis. The P2Y_11_ receptor antagonists, NF157 and NF340, inhibited ATP-evoked signalling in human endothelial cells. Vasoconstriction mediated by P2Y_12_/P2Y_13_ and P2Y_14_ receptors was characterised using the antagonists, cangrelor, ticagrelor, AR-C67085 and MRS2211 or PPTN respectively. This has yet to be backed up by receptor knockout experiments. Thus, subtype-selective antagonists and receptor knockout/knockdown have helped identify which P2Y subtypes are functionally expressed in vascular smooth muscle and endothelial cells and the effects that they mediate.

## Introduction

P2X and P2Y purinergic receptors (P2XR, P2YR) are expressed throughout the vascular system in smooth muscle and endothelial cells [1–3], where they mediate effects such as vasoconstriction, vasodilation, angiogenesis and vascular remodelling [4–7]. Their endogenous agonists, the purine and uridine nucleotides, adenosine 5'-triphosphate (ATP), uridine 5'-triphosphate (UTP), adenosine 5'-diphosphate (ADP) and uridine 5'-diphosphate (UDP), are released, both constitutively and in a regulated manner, by a variety of cell types, including perivascular nerves, endothelial cells and blood cells [1, 4]. Consequently, P2XR and P2YR have been proposed to play a number of roles in the control of vascular tone and blood pressure under physiological and pathophysiological conditions, and to be viable therapeutic targets for treatment of vascular disorders [1, 6, 7].

Early studies suggested that P2XR in smooth muscle cells mediate vasoconstriction, whereas endothelial P2YR mediate vasodilation [8–11]. However, P2YR that mediate vasoconstriction were subsequently shown to also be present in smooth muscle cells in many arteries [1, 4]. In addition, P2YR mRNA and protein are expressed in both vascular smooth muscle and endothelial cells, with the P2Y_1_R, P2Y_2_R, P2Y_4_R, P2Y_6_R and P2Y_12_R subtypes being most commonly identified [4, 12]. In order to understand how P2YR modulate vascular function it is important to know which subtypes are functionally expressed in the different cell types and what effects they mediate. For a long time, the field was hampered by a lack of potent and subtype-selective P2YR antagonists, but an increasing number of such compounds are now available [2, 13] and this has led to major advances in our understanding of purinergic signalling. For example, selective P2Y_1_R and P2Y_12_R antagonists made major contributions to the identification of the physiological role of both receptor subtypes in platelet aggregation [14] and of P2Y_1_R in gastrointestinal peristalsis [15]. Here we discuss how subtype-selective antagonists and the powerful, complementary experimental tools of receptor knockout and knockdown, have been used to identify and characterise the functions of vascular P2YR and the signalling pathways, such as Ca^2+^ mobilisation, through which they act. The use of these approaches in endothelial and smooth muscle cells is summarised in Table 1.

**Table 1:** **Selective approaches used to study roles of P2YR subtypes in vascular function.** Selective antagonists and genetic modification techniques used to study the vascular functions of each P2YR subtype are shown. +  = carried out,—= not yet carried out. Relevant references are indicated by numbers in brackets. See reference list for further details

	**Endothelium**		**Smooth Muscle**	
	**Selective antagonists**	**Knockout / knockdown**	**Selective antagonists**	**Knockout / knockdown**
**P2Y**_**1**_**R**	**MRS2179 (16–27,29,30)** **MRS2216** (28)	-	**MRS2179** (32,34) **MRS2279** (36) **MRS2500** (35)	-
**P2Y**_**2**_**R**	**AR-C118529XX (24,41–44,47)**	** + (27,44,46–51,56)**	**AR-C118529XX** (24,54)	** + (50,55,59)**
**P2Y**_**4**_**R**	-	** + (22)**	-	** + (55)**
**P2Y**_**6**_**R**	**MRS2578 (24,48,64,68,69)**	** + (48,52,61,65)**	**MRS2578 (34,61,62,63,67)**	** + (50,55,61,65)**
**P2Y**_**11**_**R**	**NF157** (29,72,73) **NF340** (70,71)	**-**	**NF340** (71)	**-**
**P2Y**_**12/13**_**R**	**Cangrelor** (24,80,81) **Ticagrelor (80,81)**	**-**	**Cangrelor** (34,35) **Ticagrelor** (79) **AR-C67085** (78) **MRS2211** (36) **MRS2395** (36)	**-**
**P2Y**_**14**_**R**	**-**	**-**	**PPTN** (63,85)	**-**

## P2Y_1_ receptors

The selective and competitive P2Y_1_R antagonist, MRS2179, has been used extensively to characterise the endothelium-dependent vasodilation induced by ADP and ATP in a wide range of blood vessels and species. Thus, it inhibited vasodilation induced by ADP in the coronary artery or coronary vascular bed of rats [16, 17], guinea-pigs [18], dogs [19] and pigs [20], the aorta of guinea-pigs [21] and mice [22], rat mesenteric bed [23], intrapulmonary artery [24] and pial arterioles [25] and dog and monkey cerebral arteries [26]. MRS2179 also shifted the ADP concentration–response curve (CRC) rightwards in the aorta of P2Y_2_R knockout mice [27], consistent with ADP not being an agonist at P2Y_2_R [2, 14]. Endothelium-dependent vasodilation of human left internal mammary arteries via P2Y_1_R was also identified using MRS2216, another P2Y_1_R antagonist [28]. In addition, MRS2179 inhibited ATP-induced Ca^2+^ influx in bovine aorta endothelial cells (BAEC) and H5V cells, which were derived from murine heart microvessel endothelium [29].

Notably, and in contrast, MRS2179 had no effect on the ATP-induced endothelium-dependent vasodilation of the rat mesenteric bed [23] and intrapulmonary artery [24] or mouse aorta [22], but it did inhibit the increase in coronary arterial blood flow elicited by ATP infusion in anaesthetised pigs by ~ 50% [30]. Thus, while ADP elicits endothelium-dependent vasodilation through P2Y_1_R, ATP appears to do so only in some cases. This is consistent with ATP being a partial agonist at P2Y_1_R and its agonist action at this receptor subtype depending upon the level of receptor expression [31]. Interestingly, MRS2179 also reduced the post-ischaemic increase in pig coronary arterial blood flow by ~ 50%, so P2Y_1_R appear to contribute to post-ischemic, coronary reactive hyperaemia and could potentially be targeted to reduce reperfusion injury that occurs during angioplasty after acute myocardial infarction [30].

MRS2179 has also been used to identify P2Y_1_R that mediate vasoconstriction, for example, in human umbilical and chorionic vessels [32]. Immunoblotting showed that P2Y_1_R were expressed in the smooth muscle rather than the endothelium and the contractions appeared to be due to downstream release of thromboxane A_2_. In addition, MRS2179 was also used to show that P2Y_1_R mediate contraction of pial vessels of spontaneously hypertensive, but not normotensive rats in vivo, although the type of cell in which the P2Y_1_R are located was not determined in this study [33]. MRS2179 also reduced the amplitude of contractions of rat intrapulmonary artery induced by MRS2365, a highly potent and selective P2Y_1_R agonist [34]. It had no effect, however, on the contractions elicited by ATP, which were instead mediated via P2X1R and P2Y_12_R, again consistent with ATP being a partial agonist at P2Y_1_R [34]. The selective and competitive P2Y_1_R antagonist, MRS2500, reduced the rise in the mean pulmonary arterial pressure induced by right atrial infusion of ADP in anaesthetised pigs [35]. MRS2500 also reduced hypoxic pulmonary vasoconstriction in this model [35], indicating that ADP and P2Y_1_R contribute to pulmonary vascular tone during acute hypoxia. Finally, the potent P2Y_1_R antagonist, MRS2279, suppressed the Ca^2+^ mobilisation evoked by MRS2365 in rat aortic smooth muscle cells in a competitive manner, but had no effect on the response to ATP [36]. Note that although P2Y_1_R knockout mice have been generated [2], the effect of this procedure on the vascular actions of ADP and ATP has not yet been reported and so cannot be compared with the actions of P2Y_1_R antagonists described above.

## P2Y_2_ receptors

The recent availability of the potent, selective and competitive P2Y_2_R antagonist, AR-C118925XX [37–40], has been a great aid in identifying and characterising responses mediated by endothelial P2Y_2_R. Thus, AR-C118925XX reduced UTP-induced, endothelium-dependent relaxation of carotid arteries from spontaneously hypertensive and normotensive Wistar-Kyoto rats [41]. It also inhibited associated endothelial signalling events, such as Ca^2+^ mobilisation evoked by ATP [42, 43] and phosphorylation of endothelial nitric oxide synthase (eNOS) and Akt in response to fluid sheer stress [44]. This is consistent with the presence of P2Y_2_R‐like immunoreactivity in carotid artery endothelial cells [42]. AR-C118925XX also inhibited UTP-, but not ATP-evoked, endothelium-dependent relaxation of rat intrapulmonary artery [24] and the UTP-evoked rise in intracellular Ca^2+^ in EAhy926 human vascular endothelial cells, shifting the agonist CRC rightwards in a parallel manner, with no decrease in the maximum response. A dissociation constant, K_B_, of 3.0 nM was determined, which is very close to that seen at recombinant human P2Y_2_R (3.7 nM) [45] (1). P2Y_2_R mRNA and protein in immunoblots [46], as well as P2Y_2_R‐like immunoreactivity [45] have also been demonstrated in EAhy926 cells.

**Fig. 1:**
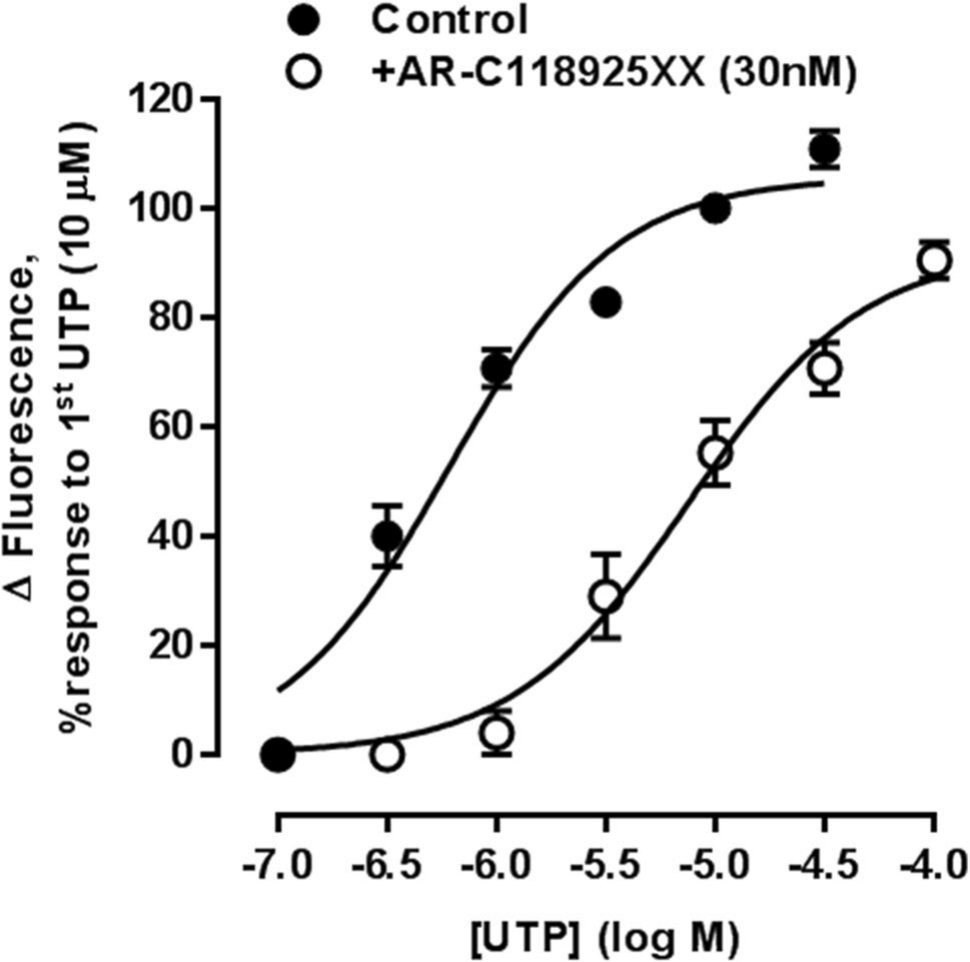
**P2Y**_**2**_** receptors mediate Ca**^**2+**^** mobilisation in EAhy926 endothelial cells.** The mean peak amplitude of responses evoked by UTP (100 nM—30 μM) when two consecutive CRC were constructed per coverslip of cells, first in the absence and then the presence of AR-C118925XX (30 nM) is shown. The data are expressed as a percentage of the response to UTP (10 µM) in the first CRC, n = 5. Vertical lines show SEM. For some points, the error bars are shorter than the height of the symbol. The curves represent the fit of the Hill equation to the data. Note that in control experiments there was no significant change in the EC_50_ value when two UTP CRC were constructed on the same population of cells in the absence of AR-C118925XX. Reproduced from Muoboghare et al. (2019), with permission from Wiley

Consistent with these data, knockdown of P2Y_2_R with siRNA suppressed Ca^2+^ mobilisation evoked by ATP and UTP in EAhy926 endothelial cells [46] and BAEC [44]. The latter study also saw a large decrease in the Ca^2+^ mobilisation, phosphorylation of eNOS and Akt and tyrosine phosphorylation of SRC kinase, PECAM-1, and VEGFR-2 induced by fluid shear stress in BAEC, indicating that P2Y_2_R play a role in initiating these signalling events. In addition, mice in which endothelial P2Y_2_R were selectively knocked out had significantly higher mean arterial blood pressure [44]. Mesenteric arteries from these mice did not vasodilate in response to an increase in flow, unlike the arteries from wild-type mice, and had less phosphorylated eNOS. The same group then reported that both AR-C118925XX and P2Y_2_R knockdown reduced substantially signalling events evoked by Yoda-1, a PIEZO-1 mechanosensitive channel agonist, in human umbilical artery endothelial cells [47]. This, together with the demonstration of ATP release by Yoda-1 [47] indicates that P2Y_2_R, activated by endogenously-released ATP, mediate the vasodilation evoked by fluid sheer stress and so lowers mean arterial blood pressure.

Knockdown of P2Y_2_R substantially reduced the UTP-evoked rise in intracellular Ca^2+^ in the human CMEC/D3 blood–brain barrier endothelial cell line [48] and upregulation of tissue factor, the initiator of the platelet coagulation cascade, induced by UTP in human coronary artery endothelial cells [49]. In contrast, P2Y_2_R knockout had no effect on endothelium-dependent vasodilation evoked by UTP in mouse aorta [27] and coronary artery [50], but it did reduce responses to ATP and ATPγS in the aorta [27] and abolished relaxations evoked by the P2Y_2_R agonist, UTPγS, in the coronary artery [50]. Selective deletion of endothelial P2Y_2_R also produced a moderate rightwards shift of the ATPγS and UTPγS CRC in the aorta [51]. These complex data suggest that in the absence of P2Y_2_R, UTP can act at other P2YR subtypes to elicit vasodilation. Consistent with this possibility, the contribution of P2Y_1_R to the action of ATP was increased in P2Y_2_R knockout mice [27]. The P2Y_4_R does not appear to be involved in the UTP response, however, as deletion had no effect [22]. In contrast, knockout of the P2Y_6_R produced a small rightwards shift in the UTP CRC in the aorta [52], indicating a possible minor role for this subtype. It is also possible that deletion of one P2YR subtype in vivo leads to upregulation of another to compensate for the loss. Unfortunately, receptor expression levels were not measured in most of these studies, although it has been found that the amount of P2Y_1_R mRNA in mouse aorta was doubled by P2Y_6_R deletion [52]. These knockout studies did not provide as clear a demonstration as would be liked of how UTP causes vasodilation, particularly in the mouse aorta. This situation is not unique, as knocking out each of the P2Y_2_R P2Y_4_R and P2Y_6_R subtypes individually had no effect against the positive inotropic action of UTP in mouse atria [53]. Full characterisation of how UTP acts may require knocking out multiple P2YR subtypes at the same time and/or using AR-C118925XX and the P2Y_6_R antagonist, MRS2578, to characterise knockout-resistant responses pharmacologically.

**Fig. 2:**
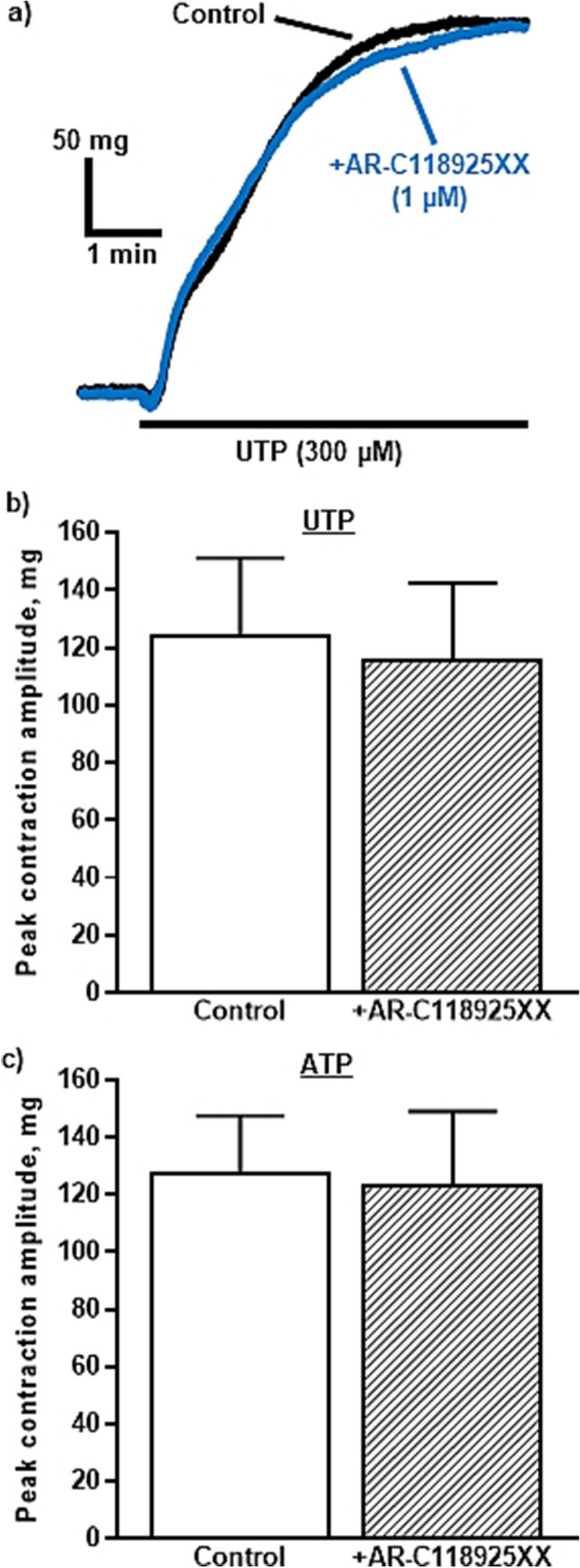
**P2Y**_**2**_** receptors do not mediate vasoconstriction of the rat intrapulmonary artery.** The superimposed traces show typical contractions of the rat isolated intrapulmonary artery evoked by UTP (300 μM) in the absence and presence of AR-C118925XX (1 μM). UTP was added as indicated by the horizontal bar. The mean peak amplitude of contractions evoked by b) UTP (300 μM) and c) ATP (300 μM) in the absence and presence of AR-C118925XX (1 $\mu$M) is shown. Vertical lines indicate SEM. n = 6 UTP, n = 5 ATP. Reproduced from Dales et al. (2022)

P2Y_2_R can also mediate vasoconstriction in vessels at resting tone, shown by the fact that AR-C118925XX abolished ATP-induced contractions of rat pulmonary veins [54]. In contrast, a high concentration of AR-C118925XX had no effect on vasoconstriction of rat intrapulmonary arteries elicited by UTP or ATP (Fig. 2), even though P2Y_2_R mRNA was extracted from endothelium-denuded tissues [24]. A ten-fold higher concentration also did not inhibit UTP- or ATP-evoked contractions of the rat tail artery (Dales, Drummond and Kennedy, unpublished observations). Similarly, P2Y_2_R knockout had no effect on UTP-evoked contractions of the mouse aorta [55] or coronary artery [50], but aortic responses to ATPγS were greatly inhibited and coronary contractions to UTPγS were abolished. Similar to the vasodilation data above, this suggests that UTP can act at other P2YR subtypes to elicit vasoconstriction and this was confirmed by the virtual abolition of UTP-evoked contractions of the aorta of P2Y_6_R knockout mice [55]. Note that responses in the same tissues to ATPγS were unaffected by deletion of the P2Y_6_R, indicating the presence of functional P2Y_2_R, so it is not clear why UTP did not act at them to evoke contraction.

Knockout of P2Y_2_R has revealed roles in cardiovascular disorders that are not directly related to their effect on vascular tone. Selectively deleting endothelial P2Y_2_R in ApoE^−/−^ mice, a model of atherosclerosis, greatly reduced the number of atherosclerotic, fatty streak lesions in the aorta [51, 56]. This was associated with reduced endothelial expression of vascular cell adhesion molecule-1, which plays an important role in adhesion of leukocytes to endothelial cells and subsequent transendothelial migration, decreased transendothelial migration of monocytes and lower levels of the inflammatory cytokine, lymphotoxin α. Deleting P2Y_2_R also shifted the atherosclerotic plaque from an inflammatory phenotype to a more stable form. Based on these and other data, the authors hypothesised that endothelial injury in the early stages of atherosclerosis causes local release of nucleotides, which act at P2Y_2_R to produce lymphotoxin α, which in turn upregulates vascular cell adhesion molecule-1 expression, so promoting inflammation. Consistent with this hypothesis, the plasma levels of ATP and ADP were higher in atherosclerotic patients than in control subjects in whom there was no clinical evidence of peripheral artery disease [57]. Low expression of ecto-nucleoside triphosphate diphosphohydrolase (CD39), which dephosphorylates tri- and diphosphate nucleotides [58], were associated with disease progression. Thus, pharmacological blockade of P2Y_2_R is a potential novel therapy for inhibiting the development of atherosclerosis.

A role for P2Y_2_R in vascular injury is also indicated by the large decrease of neointimal hyperplasia induced by injury caused by placement of a cuff around the femoral artery in P2Y_2_R-knockout compared to wild-type mice [59]. Conversely, neointimal hyperplasia was greatly increased in transgenic rats overexpressing the P2Y_2_R, producing almost total luminal stenosis.

## P2Y_4_ receptors

The lack of a selective antagonist means that there are no pharmacological data on the functions of vascular P2Y_4_R. P2Y_4_R knockout had no effect on the endothelium-dependent relaxation [22] or on the vasoconstriction [55] of mouse aorta evoked by UTP or UDP. It did, however, partially inhibit UTP-induced migration and proliferation of mouse cardiac endothelial cells and abolished their secretion of PDGF-B [60]. The knockout mice displayed fewer cardiac microvessels during development than wild-type animals, indicating that the P2Y_4_R may play a role in angiogenesis.

## P2Y_6_ receptors

The selective, non-competitive P2Y_6_R antagonist, MRS2578, has been available since 2004 and has contributed greatly to our knowledge and understanding of the roles of vascular P2Y_6_R. MRS2578 had no effect on mean arterial blood pressure [61], UTP-evoked contractions of pig pancreatic [62] and coronary [63] arteries or the mobilisation of intracellular Ca^2+^ induced by UDP in human CMEC/D3 endothelial cells [48]. It did, however, inhibit contractions of the rat intrapulmonary artery at resting tone elicited by UDP [34] and UTP (Mitchell and Kennedy, unpublished observations), whilst when tone was raised, endothelium-dependent relaxations to UDP, but not UTP, were reduced [24]. Thus, in this artery at least, UDP can induce vasoconstriction and vasodilation via smooth muscle and endothelial P2Y_6_R, respectively. Interestingly, the endothelium-dependent vasodilation of the aorta elicited by UDP was greater in obese rats compared with controls and the difference was eliminated by MRS2578, suggesting that endothelial P2Y_6_R were upregulated in obesity [64].

Conflicting data have been reported for the effects of P2Y_6_R knockout on vasodilation of the mouse aorta. One study, found that it caused a large rightwards shift in the UDP CRC and a small shift in the UTP CRC [52], whereas another saw no change in the UDP-evoked relaxations [61]. The reason for this difference is not known. Likewise, the UDP-evoked rise in intracellular Ca^2+^ in CMEC/D3 endothelial cells, although unaffected by MRS2578, was reduced by knockdown of P2Y_6_R and P2Y_2_R knockdown reduced the response by a similar extent [48]. Two separate reports concur, however, that P2Y_6_R knockout had no effect on diastolic, systolic and mean arterial blood pressure [61, 65], consistent with the lack of effect of MRS2578.

In contrast, P2Y_6_R knockout abolished contractions of mouse aorta evoked by UDP and UTP [55], of coronary artery evoked by UDP [50] and of mesenteric artery elicited by UDP, UTP and UDPβS [65]. In cultured mouse mesenteric artery smooth muscle cells, activation of the small G protein, RhoA, by UDP, UTP and UDPβS and UDP-induced Ca^2+^ mobilisation and phosphorylation of p38, ERK, JNK, myosin light chain and myosin light chain phosphatase at ser-696 and ser-853, were all abolished or greatly decreased by P2Y_6_R knockout [65]. In contrast, Ca^2+^ mobilisation evoked by UTP was barely affected and that to ATP was unaffected [65]. Ca^2+^ mobilisation induced by the P2Y_6_R agonist, PUDP, in aortic smooth muscle cells was also abolished by knockout of the P2Y_6_R [61]. Thus, P2Y_6_R clearly mediate vasoconstriction of mouse aorta.

Smooth muscle P2Y_6_R contribute to the development of myogenic tone that is evoked by a stepped increase in vascular perfusion pressure, as knockout of the receptor reduced this response by about half [65]. MRS2578 produced a similar decrease. In contrast, deleting the ecto-nucleotidase, CD39, and so inhibiting nucleotide dephosphorylation to increase extracellular nucleotide concentration, potentiated the myogenic tone and contractions evoked by exogenous UDP and UTP [55]. The myogenic tone that develops during the chronic heart failure induced by coronary artery ligation was also substantially inhibited by deletion of the P2Y_6_R, as was angiotensin II-induced hypertension [65]. In addition, P2Y_6_R knockout and MRS2578 inhibited angiotensin II-induced hypertension and it was demonstrated that this was because P2Y_6_R form stable heterodimers with AT1 angiotensin II receptors, which mediate a rise in blood pressure, vascular remodelling, oxidative stress, and endothelial dysfunction [61]. Induction of hypertension by angiotensin II also caused a decrease in the vascular expression and activity of CD39, which would reduce the breakdown of endogenous UDP and so potentiate its actions [66]. Thus, the AT1/P2Y_6_R dimer is a novel potential target for treating angiotensin II-related hypertension. MRS2578 also reduced vasoconstriction of the mouse aorta induced by UDP or angiotensin II [67], so this dimer and/or cross-talk between the two receptors, may be common within the vascular system.

P2Y_6_R have also been proposed to play a role in the development of atherosclerosis [68]. The P2Y_6_R was upregulated in endothelial cells of the aorta of low-density lipoprotein receptor-deficient mice that had been fed a high-cholesterol diet to induce atherosclerosis and global knockout of the receptor greatly reduced aortic atherosclerotic lesions. Deleting the P2Y_6_R also reduced the amount of lipid and number of macrophages present in plaques and increased the number of smooth muscle cells and the collagen content [68]. Aortic expression of vascular cell adhesion molecule-1, which plays an important role in inflammation-associated adhesion and the transendothelial migration of leukocytes, including macrophages, and production of the cytokine, IL-6, were also reduced. So, like P2Y_2_R, P2Y_6_R appear to contribute to atherosclerosis by promoting inflammation and the development of aortic plaques and their pharmacological blockade is another potential novel therapy for treating this disorder. Finally, P2Y_6_R mediates angiogenesis in cultures of human vascular endothelial cells and pericytes, as MRS2578 reduced the formation of tubules induced by the dinucleotide agonist, uridine adenosine tetraphosphate [69].

## P2Y_11_ receptors

At present there have been only a few functional studies on human vascular P2Y_11_R. In human umbilical vein and coronary artery endothelial cells, ATP inhibited the phosphorylation of JNK that was induced by IL-1β- and this effect of ATP was almost abolished by the P2Y_11_R antagonist, NF340 [70]. In addition, NF340 also inhibited the release of ATP from human umbilical vein endothelial cells and the decrease in human coronary artery smooth muscle cell proliferation induced by the P2Y_11_R agonist, NF546 [71]. Another P2Y_11_R antagonist, NF157, however, had no effect on ATP-induced Ca^2+^ influx in murine coronary microvessels and BAEC [29], indicating that ATP did not act at P2Y_11_R to produce this effect.

Blocking P2Y_11_R has been proposed to be a potential therapeutic strategy for treating atherosclerosis, as in human primary aortic endothelial cells, NF157 reduced attachment of monocytes, expression of E-selectin and vascular cell adhesion molecule-1, production of reactive oxygen species, IL-6 and TNF-α and activation of MAPK p38 induced by oxidised low-density lipoprotein, all of which contribute to this disease [72]. Similarly, NF157 inhibited enzyme-modified oxidised low-density lipoprotein-induced endothelial inflammation, monocyte accumulation and reduction of vasoreactivity in human tissue-engineered blood vessels comprising vascular endothelial and smooth muscle cells and fibroblasts [73].

Uniquely for P2YR, the *P2RY11* gene has not been identified in the genomes of mice and rats [74–76], which has greatly hindered the study of vascular P2Y_11_R. Note, however, that several reports have been published that claim to show pharmacological and immunoblotting evidence for the expression of a P2Y_11_-like receptor in murine tissues and cells [75], including blood vessels [71, 77]. This This could possibly reflect non-selective actions of NF157, NF340, and NF546 at P2YR subtypes other than the P2Y_11_R or perhaps at an as yet unidentified receptor that has low sequence homology with the human P2Y_11_R [75].

## P2Y_12_ and P2Y_13_ receptors

P2Y_12_R and/or P2Y_13_R can mediate vasoconstriction as contractions of endothelium-denuded human internal mammary [78, 79] and mouse aorta [79] and pericardial fat arteries [79] evoked by the P2Y_1_R, P2Y_12_R and P2Y_13_R agonist, 2-meSADP, were inhibited by the non-selective P2Y_12_/P2Y_13_R antagonists, AR-C67085 [78] and ticagrelor [79]. Similarly, another non-selective P2Y_12_/P2Y_13_R antagonist, cangrelor (also known as AR-C69931MX), abolished contractions of the rat intrapulmonary artery evoked by ADP [34]. In the same study, cangrelor also reduced contractions elicited by ATP in a concentration-dependent manner (3**a,b**). This cangrelor-sensitive component of the response to ATP was suggested to require dephosphorylation of ATP to ADP by CD39. The cangrelor-resistant component was virtually abolished by the P2X1R antagonist, NF449 (3**c,d**), revealing that ATP acts here via P2X1R and P2Y_12_R. Cangrelor also inhibited the rise in mean pulmonary arterial pressure induced by ADP and hypoxic pulmonary vasoconstriction in anaesthetised pigs [35]. Thus, P2Y_12_R contribute to pulmonary vascular tone during acute hypoxia. In contrast, another P2Y_12_R antagonist, MRS2395, and the P2Y_13_R antagonist, MRS2211, had no effect on Ca^2+^ mobilisation evoked by 2-meSADP in rat aortic smooth muscle cells [36], indicating that P2Y_12_R and P2Y_13_R do not contribute to this response. Similarly, cangrelor had no effect on the ATP-evoked, endothelium-dependent vasodilation of the rat intrapulmonary artery, but potentiated the responses elicited by ADP [24], presumably due to inhibition of the counteractive vasoconstriction induced by ADP via smooth muscle P2Y_12_R and/or P2Y_13_R. These pharmacological data have yet to be backed up by receptor knockout experiments, but nonetheless, smooth muscle P2Y_12_R and/or P2Y_13_R appear to mediate vasoconstriction.

**Fig. 3:**
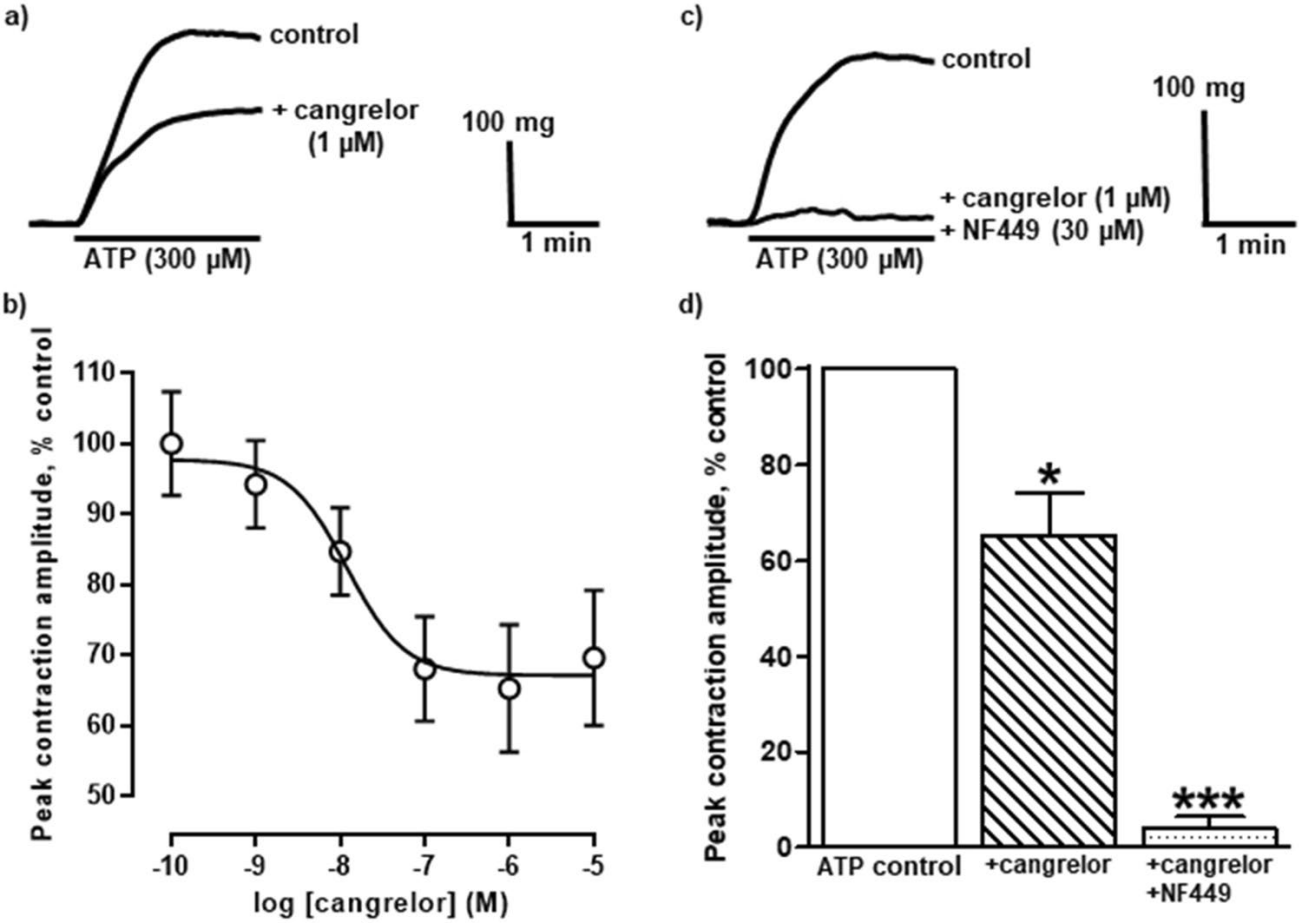
**P2X1 and P2Y**_**12**_** receptors mediate vasoconstriction of the rat intrapulmonary artery.** a) The superimposed traces show typical contractions of the rat isolated intrapulmonary artery evoked by ATP (300 $\mu$M) in the absence (upper trace) and presence (lower trace) of cangrelor (1 $\mu$M). ATP was applied as indicated by the solid bars. b) The mean peak amplitude of contractions evoked by ATP (300 $\mu$M) in the presence of cangrelor (0.1 nM—10 $\mu$M) is shown. Vertical lines show SEM, (n = 4–6). The curve represents the fit of the Hill equation to the data. c) The superimposed traces show typical contractions evoked by ATP (300 $\mu$M) in the absence (upper trace) and presence (lower trace) of cangrelor (1 $\mu$M) plus the P2X1R antagonist, NF449 (30 $\mu$M). d) The mean peak amplitude of contractions evoked by ATP (300 $\mu$M) in the presence of cangrelor (1 $\mu$M) (cross-hatched column) (n = 4) and cangrelor (1 $\mu$M) plus NF449 (30 $\mu$M) (speckled column) (n = 6) are shown. * *P* < 0.05, ***P < 0.001 for responses to ATP in the presence of antagonists compared to in their absence. Reproduced and modified from Mitchell et al. (2012), with permission from ASPET

In human umbilical vein [80] and pulmonary microvascular [81] endothelial cells ticagrelor and clopidogrel inhibited a variety of processes that underlie LPS-induced dysfunction, such as increased production of TNF-α, IL-1β and IL-6, decreased levels of nitric oxide, eNOS and p-Akt, decreased cell migration, increased apoptosis, decreased cell viability and increased endothelial cell layer permeability. Thus, P2Y_12_R antagonists could potentially be used to target endothelial cell inflammation and dysfunction. Indeed, this is consistent with the report that long-term administration of clopidogrel reduced inflammation in pigs [82]. A point to note, however, is that although ticagrelor acts directly at the P2Y_12_R, clopidogrel, which is in widespread clinical use, has no direct effect. Instead, it is a liver-activated prodrug and its metabolic products interact irreversibly with the P2Y_12_R [83, 84]. Thus, it remains to be clarified how clopidogrel produced the same effects as ticagrelor in these studies.

## P2Y_14_ receptors

UDP and UDP-glucose are both agonists at the P2Y_14_R and the P2Y_14_R antagonist, PPTN, inhibited contractions of pig pancreatic [85] and coronary [63] arteries induced by UDP-glucose and the P2Y_14_R agonist, MRS2690. Interestingly, the responses in the former, but not the latter tissue were reduced by physical removal of the endothelium and the contractions were dependent, at least in part, on endothelial production of thromboxane A_2_, prostaglandin F_2α_ and endothelin-1 [63]. In contrast, a high concentration of UDP-glucose did not elicit vasoconstriction of the rat intrapulmonary artery, suggesting that P2Y_14_R do not mediate vasoconstriction in this tissue [34].

## Discussion

The data discussed above, obtained using subtype-selective antagonists and receptor knockout or knockdown, show that most P2YR subtypes are functionally expressed in vascular smooth muscle cells and/or endothelial cells. The most commonly studied and best characterised actions relate to modulation of vascular tone and the associated intracellular signalling pathways, i.e. endothelium-dependent vasodilation mediated by P2Y_1_R, P2Y_2_R and P2Y_6_R and vasoconstriction mediated by P2Y_1_R, P2Y_2_R, P2Y_6_R, P2Y_12_ and/or P2Y_13_ and P2Y_14_R. These are proposed to contribute to post-ischemic coronary reactive hyperaemia (P2Y_1_R), hypoxic pulmonary vasoconstriction (P2Y_1_R, P2Y_12_R), vasodilation in response to flow sheer stress (P2Y_2_R) and the myogenic tone evoked by a stepped increase in vascular perfusion pressure or which develops during chronic heart failure (P2Y_6_R). A role for P2Y_2_R in neointimal hyperplasia induced by vascular injury has also been suggested and several studies have provided evidence that P2Y_2_R, P2Y_6_R and P2Y_11_R contribute to the development of atherosclerosis. Thus, P2YR clearly play a variety of roles in the control of vascular function under physiological and pathophysiological conditions and are potential therapeutic targets for treatment of vascular disorders.

Whilst great advances in our knowledge and understanding have clearly been made, there are, nonetheless, factors that hamper further progress. First, we still lack potent, competitive and selective antagonists for many of the P2YR subtypes. The development of such drugs always leads to major advances in our understanding of receptor signalling. Selective, metabolically-stable agonists would also be useful. The endogenous agonists, ATP, ADP, UTP and UDP, are neither subtype-selective nor metabolically-stable. Dephosphorylation by ecto-enzymes not only reduces their potency, but can also produce metabolites that are active at the same or other receptors, i.e. ADP produced from ATP, adenosine from ATP and ADP, and UDP from UTP. Similarly, commercially-available nucleotides tend not to be 100% pure and may contain small amounts of related nucleotides [31], which can also complicate interpretation of the data in the same way. In addition, ecto-nucleoside diphosphokinases can catalyse formation of triphosphates from diphosphates [86]. Agonist metabolism and/or activation of multiple types of receptor produces shallow agonist CRC that may not reach a plateau [87], which indeed was seen for ATP-, ADP-, UTP- and UDP-evoked relaxation of the rat intrapulmonary artery [24]. Consistent with the influence of breakdown, contractions of mouse aorta evoked by UDP and UTP were significantly potentiated by reducing their breakdown by knocking out CD39 and the slopes of their CRC were greatly increased [55].

P2YR knockout and knockdown are powerful experimental tools and have made a great contribution to our understanding of the functions of vascular P2YR, but in some cases the data obtained have been complex or inconsistent, for instance, P2Y_2_R [27, 50, 51] and P2Y_6_R [48, 52, 61, 65]. A possible explanation is that inhibiting the expression of one P2YR subtype in vivo leads to compensatory upregulation of another, for example, the amount of P2Y_1_R mRNA in mouse aorta was doubled by P2Y_6_R deletion [52]. To overcome this limitation, conditional receptor knockout would ideally be used, which could be extended to include knockout of multiple subtypes in the same animal. In addition, the actions of subtype-selective antagonists could also be determined in these animals. Such an intensive approach would be costly, but would likely resolve the reported inconsistencies and complexities and provide a fuller characterisation of individual receptor subtype function.

Another limitation to consider is that although some actions have been studied in depth, e.g. P2Y_2_R function and signalling in carotid artery endothelial cells [42], few studies have provided a more global view of P2YR vascular function by investigating the effects mediated by more than one subtype in an individual artery or vein. At present, the rat intrapulmonary artery is perhaps the best example of where this has been done. Thus, initial studies employing the non-selective antagonists, suramin and PPADS [88, 89], were developed by using the selective antagonists and demonstrated that P2Y_1_R, P2Y_2_R and P2Y_6_R mediate endothelium-dependent vasodilation, whilst P2Y_1_R, P2Y_6_R and P2Y_12_R and/or P2Y_13_R, but not P2Y_14_R, together with P2X1R, mediate vasoconstriction [24, 34, 90]. Questions remain, however. For example, what mediates the component of UTP-evoked vasodilation that is not blocked by a high concentration of AR-C118925XX? How does ATP elicit vasodilation, as P2Y_1_R, P2Y_2_R, P2Y_12_R, P2Y_13_R and adenosine receptors do not appear to be involved? What underlies UTP-evoked vasoconstriction, as this response is unaffected by AR-C118925XX? It may be that when one subtype is blocked pharmacologically, nucleotides can act at another subtype and this potential mechanism could be investigated by coapplying two or more of the antagonists at the same time, as performed in rat intrapulmonary artery [34]. Nonetheless, it is clear that there is still great scope for using subtype-selective antagonists and receptor knockout and knockdown to provide a fuller characterisation of the roles of P2YR in vascular function under physiological and pathophysiological conditions and great potential for targeting them therapeutically to treat vascular disorders.

A final potential cautionary note is the complexity of purinergic receptor signalling. The large number of different P2YR and P2XR, their widespread expression (and often coexpression) in so many tissues and cell types throughout the body and the presence of multiple endogenous agonists, which have many (sometimes opposing) actions, together comprise a signalling system, which is so extensive and heterogeneous that it might potentially limit or prevent the development of useful therapeutic agents. There are, however, clear examples where these factors have not been an insurmountable hindrance. For example, platelets express both P2Y_1_R and P2Y_12_R, as well as P2X1R, but this did not prevent the development of clopidogrel and other subsequent selective P2Y_12_R antagonists for the treatment of thrombosis, acute coronary syndrome and coronary artery disease [1–3]. Other pertinent success stories can also be seen beyond purinergic signalling. Notably, the adrenoceptor family comprises nine GPCR subtypes (α_1A_, α_1B_, α_1D_, α_2A_, α_2B_, α_2C_, β_1_, β_2_, β_3_) [13], which are expressed in most tissues and cell types in the body. Nonetheless, selective β_1_-adrenoceptor antagonists, such as atenolol, and non-selective β_1_/β_2_-adrenoceptor antagonists, such as propranolol, are widely prescribed for the treatment of hypertension, angina, class II dysrhythmia, glaucoma and migraine and to relieve symptoms of anxiety [91]. Similarly, selective β_2_-adrenoceptor agonists, such as salbutamol and salmeterol, are widely used to treat asthma [91] and less commonly, can also be administered to delay premature labour in pregnant women [91]. Selective α_1_-adrenoceptor antagonists, such as prazosin, were once frontline anti-hypertensive agents and are still prescribed in certain cases [91]. More recently, α_1_-adrenoceptor antagonists, such as doxazosin and tamsulosin, were introduced as treatment for benign prostatic hyperplasia [91]. Thus, although the complexity of purinergic receptor signalling makes the characterisation of the actions that they mediate more difficult, it need not prevent development of new therapeutic agents. The crucial factor and key to success is synthesis of further selective ligands for the P2YR subtypes, which will greatly increase the potential for the development of new pharmacotherapeutic strategies.

## Data Availability

Not applicable.

## Abbreviations

ADP: Adenosine 5'-diphosphate
ATP: Adenosine 5'-triphosphate
BAEC: Bovine aorta endothelial cells
CD39: Ecto-nucleoside triphosphate diphosphohydrolase
CRC: Concentration-response curves
eNOS: Endothelial nitric oxide synthase
P2XR: P2X receptor; P2YR—P2Y receptor
UDP: Uridine 5'-diphosphate
UTP: Uridine 5'-triphosphate
